# Level of cardiovascular disease knowledge, risk perception and intention towards healthy lifestyle and socioeconomic disparities among adults in vulnerable communities of Belgium and England

**DOI:** 10.1186/s12889-022-12608-z

**Published:** 2022-01-29

**Authors:** Hamid Yimam Hassen, Mark Bowyer, Linda Gibson, Steven Abrams, Hilde Bastiaens

**Affiliations:** 1 Department of Family Medicine and Population Health, Faculty of Medicine and Health Sciences, University of Antwerp, Doornstraat 331 Wilrijk, 2610 Antwerp, Belgium; 2 Institute of Health and Allied Professions School of Social Sciences, Nottingham Trent University, Nottingham, NG1 4FQ UK; 3 Global Health Institute, Faculty of Medicine and Health Sciences, University of Antwerp, 2610 Antwerp, Belgium; 4 Interuniversity Institute for Biostatistics and statistical Bioinformatics, Data Science Institute, Hasselt University, 3590 Diepenbeek, Belgium

**Keywords:** Cardiovascular, Knowledge, Risk perception, Socioeconomic variation, Belgium, England

## Abstract

**Background:**

The burden of cardiovascular diseases (CVDs) greatly varies between and within countries. Low- and middle-income countries (LMICs) and vulnerable communities of high-income countries (HIC) share disproportionately higher burden. Evidence is limited on the level of CVD knowledge and risk perception in vulnerable communities. Hence, in this study, we assessed the level of CVD knowledge, risk perception and change intention towards physical activity and healthy diet among vulnerable communities in Antwerp, Belgium and Nottingham, England. Furthermore, we investigated the socioeconomic disparities particularly in the Antwerp setting.

**Method:**

A cross-sectional study was performed among 1,424 adults (958 in Antwerp and 466 in Nottingham) aged 18 or older among selected vulnerable communities. Districts or counties were selected based on socioeconomic and multiple deprivation index. A stratified random sampling was used in Antwerp, and purposive sampling in Nottingham. We determined the level of CVD knowledge, risk perception and intention towards a healthy lifestyle in Antwerp and Nottingham using a percentage score out of 100. To identify independent socioeconomic determinants in CVD knowledge, risk perception, intention to PA and healthy diet, we performed multilevel multivariable modeling using the Antwerp dataset.

**Results:**

The mean knowledge percent score was 75.4 in Antwerp and 69.4 in Nottingham, and only 36.5% and 21.1% of participants respectively, had good CVD knowledge (scored 80% or above). In the multivariable analysis using the Antwerp dataset, level of education was significantly associated with (1) CVD knowledge score (Adjusted β = 0.11, 95%CI: 0.03, 0.18), (2) risk perception (0.23, 95%CI: 0.04, 0.41), (3) intention to physical activity (PA) (0.51, 95%CI: 0.35, 0.66), and (4) healthy diet intention (0.54, 95%CI: 0.32, 0.75). Furthermore, those individuals with a higher household income had a better healthy diet intention (0.44, 95%CI: 0.23, 0.65). In contrast, those who were of non-European origin scored lower on intention to have a healthy diet (-1.34, 95%CI:-2.07, -0.62) as compared to their European counterparts. On average, intention to PA was significantly higher among males (-0.43, 95%CI:-0.82, -0.03), whereas females scored better on healthy diet intention (2.02, 95%CI: 1.46, 2.57).

**Conclusions:**

Knowledge towards CVD risks and prevention is low in vulnerable communities. Males have a higher intention towards PA while females towards a healthy diet and it also greatly varies across level of education. Moreover, those born outside Europe and with low household income have lower healthy diet intention than their respective counterparts. Hence, CVD preventive interventions should be participatory and based on a better understanding of the individuals’ socioeconomic status and cultural beliefs through active individual and community engagement.

**Supplementary Information:**

The online version contains supplementary material available at 10.1186/s12889-022-12608-z.

## Introduction

Cardiovascular diseases (CVDs) take a large share to the growing public health challenge of non-communicable diseases (NCDs). In 2019, over half a billion prevalent cases, 18.6 million deaths, and 393 million disability adjusted life years (DALYs) were due to CVDs [1], so being a major contributor of adult morbidity and mortality. Coherently, CVDs are the leading cause of death in Europe, with estimated 4 million deaths each year and 25% of all-cause DALYs [2]. In Belgium and the United Kingdom (UK), CVDs respectively account for 28% and 27% of all deaths, and 14.2% and 14.9% of total DALYs [2, 3].

The burden of CVDs greatly varies across countries and regions, with over three-quarters of deaths occurring in low- and middle-income countries (LMICs) [4]. Although the incidence and mortality due to CVDs is higher in LMICs [5], vulnerable communities of high-income countries (HIC) are also being affected to a large extent [6]. Socially and/or economically disadvantaged groups are disproportionately at higher risk. Socioeconomic status including income, education, occupation, living environment and access to healthcare services have all been linked with CVD incidence and mortality [6–11], indicating the impact of social and financial deprivation on CVDs and risk factors. Vulnerable populations including elderly, racial and ethnic minorities, unemployed, uninsured and under-insured populations also take a large share of the burden [12]. Hence, it is crucial to provide more attention to groups and individuals who are at greatest risk to minimize the widespread inequality in the burden of CVDs.

The effect of lifestyle including physical activity (PA), dietary habit, tobacco use, excessive alcohol consumption, and stress on CVDs is well documented [13–15]. Likewise, adopting a healthy lifestyle reduces CVD incidence and mortality at population level [16]. Studies showed that knowledge of behavioral risks is the central element of lifestyle change and individuals who perceive themselves at higher risk of CVDs are more likely to adopt a healthy lifestyle [17–21]. Therefore, measuring knowledge, risk perception and intention towards a healthy lifestyle is essential to develop and implement targeted public health interventions [21–23]. Evidence is limited on differences in knowledge and risk perception and their role in variation of CVD burden in vulnerable communities. As a result, it is pivotal to investigate the level and socioeconomic disparities in knowledge on CVDs, risk perception and intention to change lifestyle that could play a role in the observed higher burden in vulnerable communities. Examining disparities within the vulnerable communities could guide in prioritization of intervention components and reach out to more disadvantaged groups.

A multi-country CVD prevention implementation project named ‘SPICES’ – Scaling-up Packages of Interventions for Cardiovascular diseases in Europe and Sub-Saharan Africa aimed to reduce CVD risks in LMICs and vulnerable populations of HICs. The project is being conducted in selected areas of Belgium, England, France, South-Africa and Uganda. This particular study aimed to investigate the level of knowledge, risk perception and intention to healthy lifestyle among adults living in selected vulnerable communities of Nottingham (England) or Antwerp (Belgium) as well as to identify socioeconomic disparities particularly in Antwerp. The results from this study provide evidence on which segments of the population should be targeted for the intervention and to inform public health professionals working on CVD prevention to give more emphasis for high-risk vulnerable groups.

## Method

### Study settings

We used the data collected in the baseline surveys of the SPICES project, particularly Antwerp and Nottingham. Details of the methods particularly the setting and procedures in the city of Antwerp is described elsewhere [24]. Nottingham is a city in Nottinghamshire, England, which has a total population around 330,000. In general, the SPICES project in Europe aimed to reach vulnerable communities applying the principle of ‘proportionate universalism’ [25], targeting vulnerable districts or counties rather than individual level vulnerability. In Antwerp, two districts (Deurne Noord and Borgerhout Intramuros) were selected based on a higher socioeconomic deprivation index (SDI), lower access to primary healthcare, a higher density of households with social support, and a higher density of elderly inhabitants. The SDI was calculated using the following indicators: i) the share of long-term unemployed jobseekers in the occupational age population (18-64 yrs); ii) the share of occupational age population that receives social support; and iii) the share of taxpayers with net taxable income of less than 10,000 euro per year. The score ranges from 0 (lowest) to 16 (highest), the higher indicating more vulnerability. Similarly in Nottingham, socioeconomically disadvantaged, lower socio-economic status (SES) postcodes were identified using the overall Multiple Deprivation Index (MDI) [26]. The MDI consists of average household income, employment rate, health and disability, education and skills, housing, living environment and crime rate [26].

### Study participants and procedure

The study procedure in the Antwerp setting is available elsewhere [24]. A survey was conducted on a random sample of residents in selected districts using probability proportional to size (PPS) sampling. Registered residents who were aged 18 to 75 years were eligible. To use a matched substitution technique [27], for each initially selected participant, three matched substitutes were identified in advance within the same age group, of the same sex and geographical location. Out of 1,512 matched sets invited (4 individuals with same age group, sex and geographic location), 975 had at least one respondent. Of which, 17 questionnaires were excluded due to either the participant was not eligible or missing responses in the required items, making the effective sample size for this analysis to be 958. In Nottingham, participants were recruited using purposive sampling through existing community groups, organizations and venues, and employees in the workplace. All residents aged 18 to 75 and living in the selected study areas were eligible to participate in the study. As a result, a total of 483 participants completed the baseline survey, of which 17 had missing information in all the required items leading to an effective sample size of 466 individuals. In total, 1,424 participants were included in the current analysis.

### Data collection procedure and instruments

In the Antwerp setting, the survey invitation was sent through post and participants were requested to respond either via pre-paid post or online through the link provided in the information sheet. In Nottingham, face to face interviews were conducted between April 2019 and March 2020.

To assess participants’ knowledge, risk perception and intention to change PA and dietary habits, the Attitudes and Beliefs about Cardiovascular Disease (ABCD) Risk Questionnaire [28] was employed in both settings. The questionnaire was modified and translated in Dutch for the Antwerp setting with few additional questions (see Additional file 1). The questionnaire has two main components, the knowledge and risk scale. The risk scale further comprised three sub-scales namely, risk perception, intention to PA, and healthy diet intention. The Dutch (Flemish) version of the questionnaire was validated and showed good psychometric properties, with reliability coefficient of the knowledge, risk perception, intention to PA, and healthy diet intention scale of 0.75, 0.93, 0.88 and 0.84 respectively [24].

Furthermore, in Antwerp, socioeconomic characteristics of participants including age, sex, country of birth, level of education, occupation, estimated net monthly income, and family size were collected. In Nottingham, only age and sex were collected at the individual level.

### Statistical analyses

The paper form questionnaires were entered into the Research Electronic Data Capture (REDCap) tool hosted at the University of Antwerp [29]. The data collected electronically through online (Antwerp) and field tablets (Nottingham) was automatically stored to the REDCap. For data processing and analysis, we exported the .CSV file from REDCap to the free R software package, version 4.0.2 [30].

Socioeconomic variables were summarized using absolute and relative frequencies or mean/median with standard deviation (SD)/interquartile range (IQR). To remediate the unequal response rate across groups and substitution in Antwerp, we calculated the post-stratification sampling weight using the population and sample proportion within the respective sex, age group and geographical location using data from the Antwerp city administration. Weights were calculated using an iterative proportional fitting (raking) procedure [31]. Then, the survey weights were taken into consideration for further analysis.

To obtain the knowledge score, we summed up all the knowledge items and the total score was generated for each participant with a maximum of 9 for Antwerp and 8 for Nottingham (one additional item in Antwerp). Similarly, the three risk sub-scales were also summed up independently to generate total scores of risk perception (range from 7 to 28), intention to PA (6 to 24) and healthy diet intention (7 to 28 for Antwerp and 5 to 20 for Nottingham). Due to minor variation in the number of items included in each setting (for the knowledge and healthy diet intention) we rescaled the total scores to a percentage score out of 100 for comparison purposes. Then, based on Bloom’s cutoff, we categorized knowledge as good for those with a percentage score of above 80% [32] and we compared the level of CVD knowledge in two settings using a Z-test for two-independent proportions. Furthermore, risk perception, intention to PA and healthy diet intention scores were also compared across settings using an unpaired t-test.

Since individual level socioeconomic characteristics were not available for Nottingham, we used only the Antwerp dataset for multivariable regression. To examine the effect of socioeconomic characteristics on CVD knowledge, risk perception, intention to PA and healthy diet intention score, we employed a linear mixed model with 47 statistical sectors as clustering variable (i.e. a random intercept for each sector). To examine the clustering effect, we computed the Intraclass Correlation Coefficient (ICC) at the level of statistical sectors. Visual inspection of residual plots did not reveal any obvious deviations from homoscedasticity or normality. Four different regression models were used to investigate the effects of socioeconomic characteristics on each outcome measure separately. Multicollinearity between independent variables in the regression model was assessed using (generalized) variance inflation factors (VIFs). In all the analyses, a two-sided *P*-value < 0.05 was considered statistically significant.

## Results

### Socioeconomic characteristics of participants

The mean age of the respondents was 49.2 years (SD: 15.9) in Antwerp and 48.8 years (SD: 18.8) in Nottingham, with no significant difference between these settings (*P*-value = 0.241). The majority of participants (56.4%) in Antwerp and half (50.2%) in Nottingham were females. In Antwerp, 163 (17.0%) were born in non-European countries, 55.3% were married, and 13.0% attended less than 10 years of education. Above a quarter (27.1%) earned a net household monthly income below 1,667 Euro. Details of the socioeconomic characteristics of the respondents in Antwerp are presented in Table 1.

**Table 1:** Socioeconomic characteristics of study participants in Antwerp (*n* = 958)

**Socioeconomic characteristics**	**Mean (SD)**
**Age**	49.8 (15.9)
	**Frequency (%)**
**Sex**	
Male	418 (43.6)
Female	540 (56.4)
**Country of birth** (*n* = 522)	
Europe	794 (82.9)
Outside Europe	163 (17.1)
**Marital status** (*n* = 521)	
Single	274 (28.7)
Married	528 (55.3)
Widowed	37 (3.9)
Divorced	116 (12.1)
**Highest years of education** (*n* = 513)	
<10 years in school	123 (13.0)
10-12 years in school	236 (25.0)
13-14 years of school	146 (14.4)
15 years	252 (26.7)
16 years and above	188 (19.9)
**Household net monthly income (€)** (*n* = 507)	
< 833	34 (3.7)
833 - 1.667	217 (23.4)
1.667 - 2.500	284 (30.7)
2.500 - 3.333	167 (18.0)
3.333 - 4.167	106 (11.4)
> 4.167	118 (12.7)

*SD *Standard deviation

### Level of CVD knowledge and risk perception in Antwerp and Nottingham

**Fig. 1:**
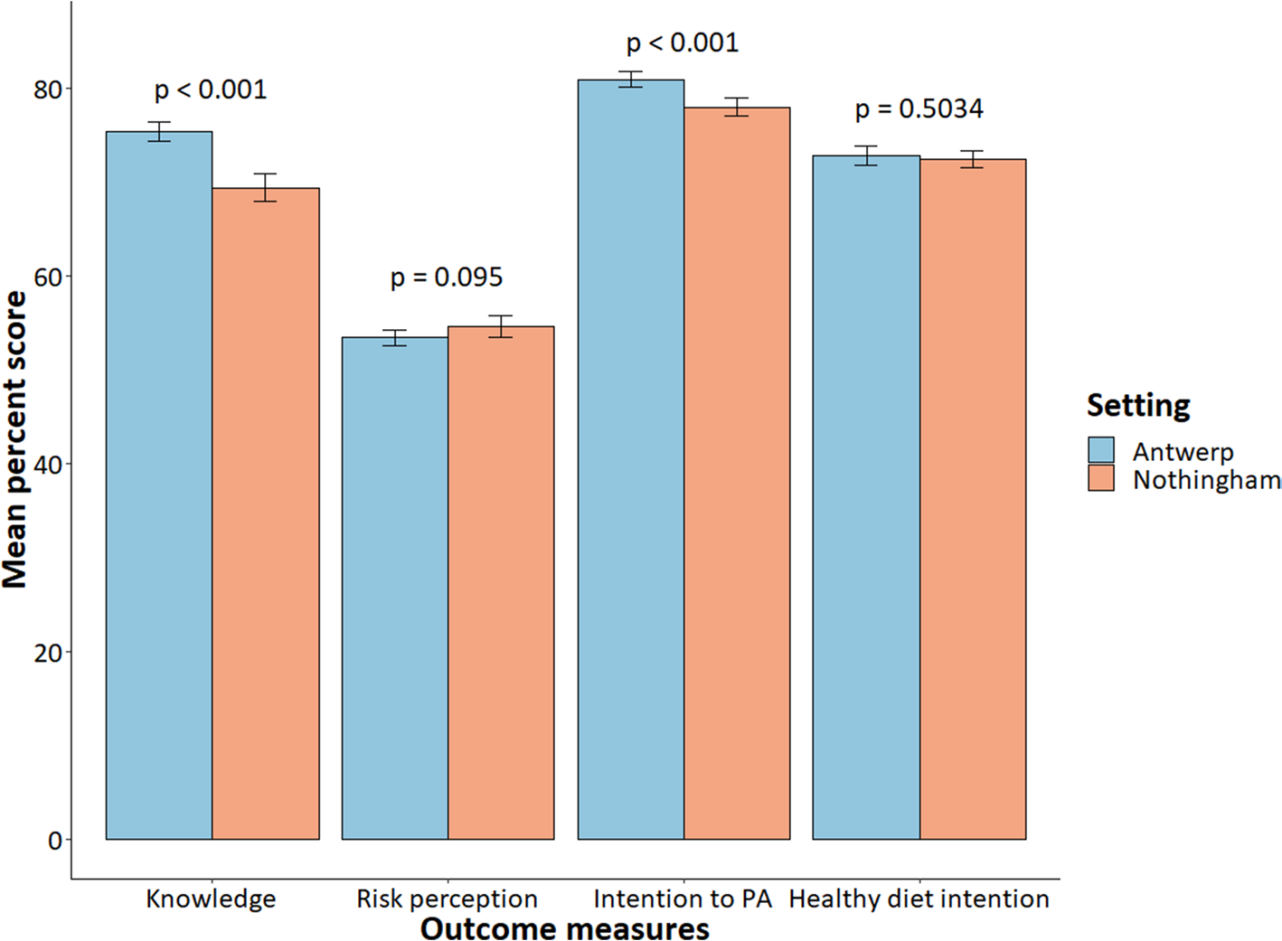
Level of CVD knowledge, risk perception, intention to PA and health diet score in Antwerp (*n* = 958) and Nottingham (466), 2020. Mean percent score: a raw score rescaled to 100

The mean knowledge percent score was 75.4 in Antwerp and 69.4 in Nottingham, and 36.5% and 21.2% of participants respectively, had good CVD knowledge (scored 80% or above). The mean risk perception score was 53.4 and 54.6 in Antwerp and Nottingham respectively, and the difference was not significant (MD = -1.2, 95%CI: -2.62, 0.21, *p* = 0.095). The PA intention score was 80.9 in Antwerp and 78.0 in Nottingham, and the difference was statistically significant (MD = 2.9, 95%CI: 1.64, 4.17, *p*<0.001). Healthy diet intention score was 72.8 in Antwerp and 72.4 in Nottingham, with no statistically significant variation (MD = 0.45, 95%CI: -0.87, 1.77, *p* = 0.5034). (Fig. 1)

In Antwerp, 23.7%, 33.9%, 5.8% and 18.1% of participants, respectively, do not know that stress, diabetes, smoking, and consumption of large amounts of alcohol are risk factors of CVDs. In Nottingham, 18.7%, 27.7% and 20.8%, respectively, do not know that stress, diabetes and consumption of large amounts of alcohol are risk factors of CVDs. Nearly two-third (65.9%) in Antwerp and 82.0% in Nottingham, respectively, were not aware of family history being a risk factor of CVDs. Moreover, 12.4% in Antwerp and 4.7% in Nottingham do not consider walking and gardening as exercises that can lower the risk of having a heart attack or stroke.

### Multivariable linear mixed model

We used four independent multilevel linear mixed models (individuals clustered within statistical sectors) to evaluate the effects of socioeconomic characteristics on knowledge, risk perception, intention to PA and healthy diet, separately, in the city of Antwerp. The ICC was 0.034, 0.013, 0.018 and 0.022 for knowledge score, risk perception, intention to PA and intention to healthy diet t respectively, indicating large proportion of variability in the outcomes is attributable to individual level differences. The multivariable regression showed that level of education was significantly associated with knowledge score (β = 0.11, 95%CI: 0.03, 0.18), risk perception (β = 0.23, 95% CI: 0.04, 0.41), intention to PA (β = 0.51, 95% CI: 0.35, 0.66), and healthy diet intention (β = 0.54, 95% CI: 0.32, 0.75), indicating those with higher level of education had a better score in these respective endpoints. A higher household income level was also significantly associated with a better healthy diet intention (β = 0.44, 95% CI: 0.23, 0.65). On average, intention to PA was significantly lower among females (β = -0.43, 95%CI: -0.82, -0.03), whereas on intention to healthy diet, females scored higher (β = 2.02, 95%CI: 1.46, 2.57). Those who were of non-European origin have lower intention towards a healthy diet compared with their European counterparts (β = -1.34 , 95% CI: -2.07, -0.62) (Table 2).

**Table 2:** Multivariable linear mixed model on the effect of socioeconomic characteristics on CVD knowledge, risk perception, intention to change of PA, and healthy diet intention in Antwerp

**Socioeconomic characteristics**	**Adjusted β (95%CI)**			
	**Knowledge**	**Risk perception**	**Intention to PA**	**Healthy diet intention**
Intercept	5.68	14.7	17.0	15.0
Age	0.004 (-0.003, 0.01)	0.03 (0.01, 0.04)***	-0.01 (-0.02, 0.001)	0.03 (-0.002, 0.05)
Sex (*female*)	0.04 (-0.15, 0.24)	0.12 (-0.240, 0.46)	-0.43 (-0.82, -0.03)**	2.02 (1.46, 2.57)***
County of birth (*outside Europe*)	0.10 (-0.04, 0.15)	-0.09 (-0.03, 0.19)	-0.01 (-0.53, 0.51)	-1.34 (-2.07, -0.62)***
Education level	0.11 (0.03, 0.18)**	0.23 (0.04, 0.41)*	0.51 (0.35, 0.66)***	0.54 (0.32, 0.75)***
Income	0.07 (-0.001, 0.15)	0.16 (-0.02, 0.34)	0.02 (-0.13, 0.17)	0.44 (0.23, 0.65)***

^*^ p < 0.05; ** p < 0.01; *** p < 0.001; PA physical activity

Interaction was assessed between age and sex, education level and income, education level and country of birth, and country of birth and income

## Discussion

CVDs, principally heart attack and stroke, continue to be the top-ranked causes of disease burden globally. It is evidenced that a significant proportion of morbidity and mortality due to CVDs can be ameliorated by targeting behavioral risks including smoking, unhealthy dietary habits, physical inactivity, alcohol consumption and stress. Nevertheless, these major risk factors are still prevalent causing a large proportion of disease burden in all regions of the world. Low-income countries and vulnerable communities living in high-income countries share a remarkably high CVD burden. Due to the superficial perception of Europe as a wealthy region, vulnerable communities living in these countries have received little attention. Targeting knowledge and risk perception of such vulnerable communities is vital to change behavior towards a healthy lifestyle, which is the cornerstone of CVD prevention. Hence, we investigated the level of CVD knowledge, risk perception and intention to healthy lifestyle among adults living in vulnerable districts of Antwerp and Nottingham. We also examined the socioeconomic disparities in these outcome measures within the vulnerable districts of Antwerp. Thus, this study provides evidence of low CVD knowledge in vulnerable communities, in which majorities in both settings have poor knowledge related to CVDs and risk factors. We also found gender and other socioeconomic variations in perceived benefit and intention towards a healthy lifestyle. Furthermore, the level of education, household income and sex highly correlated with risk knowledge and intention towards a healthy lifestyle.

In this study, only one-third of participants in Antwerp and one-fifth in Nottingham had good knowledge related to CVDs and risk factors. Although the sampling procedure varies, consistent results from both settings showed that the level of knowledge related to CVDs is low, implying the need for more targeted interventions in vulnerable communities living in high-income countries. Vulnerable communities of high-income countries are overlooked due to the superficial consideration of these countries to have a better understanding of CVDs and risk factors. Our results indicated that there is still a lot to be done to improve CVD knowledge and risk perception in developed countries. When intervention mechanisms are considered to prevent and control CVDs, it is crucial to identify groups and individuals at higher risk and therefore preventive programs should focus on those groups.

CVD knowledge score was higher in Antwerp than Nottingham. However, the variation in sampling and data collection procedure between two settings could be the main reason for the difference. In Nottingham, face to face interviews were employed using purposive sampling, which is presumed to be better in reaching more vulnerable individuals. Whereas in Antwerp the survey was on a random sample via either postal or online, in which the non-respondents might differ in socioeconomic composition and health concern leading to variation within the vulnerable communities. Studies showed that socioeconomically disadvantaged groups are relatively underrepresented in postal and online surveys [33, 34]. Socioeconomically better-off are more aware and concerned about health and are more likely to respond, which might lead to a higher knowledge score in Antwerp than Nottingham.

The multivariable analysis particularly for the Antwerp setting found that females have higher intention towards a healthy diet than males. Consistently, a study in Denmark found a significant gender difference in healthy diet intention [35]. Likewise, other studies also showed variation in perceived benefit and intention to change towards a healthy diet across gender [36, 37]. Although the intention towards a healthy diet is higher among women, the actual behavior might be determined by several aspects of the food system including the purchasing power and access to healthy food [35]. Hence, public health professionals and primary caregivers should put an effort to translate such higher intention among females to actual positive behaviors so as to prevent CVDs in vulnerable communities.

In contrast, our study showed that females have a lower intention to PA than males. A similar pattern was reported by Gavin and colleagues, which indicated males have a better intention to follow exercise recommendations than females [38]. This could be due to the gender variation in self-efficacy, social support and motivation which might lead to discrepancy in the level of physical activity [39]. Moreover, lack of access to equipment, safe and welcoming facilities, and cultural barriers for females could be the possible reasons [40, 41]. Thus, CVD preventive interventions aimed to improve physical activity should consider such pronounced gender differences to optimize intervention uptake and effectiveness. Furthermore, establishing female friendly physical activity centers could help to improve their intention as well as behavior towards physical activity [41]. Furthermore, a qualitative study to investigate an in-depth understanding on the possible reason for such gender disparities is recommended.

The predictive and causal effect of education on CVD is still controversial, meaning whether the correlation is due to reverse causality or confounding with other determinants remains unclear. In our study, level of education was significantly related to CVD knowledge, risk perception and healthy lifestyle intention. Similar studies also reported a positive effect of education on knowledge about CVD risk factors and prevention [42–44], implying special attention needs to be provided for those with lower levels of education. Furthermore, studies also demonstrated that a higher education level is associated with better dietary behavior [45]. Thus, level of education has an independent effect on CVD knowledge and intention to change, which are initial stages of behavioral change models.

The present study showed that healthy diet intention significantly varies across socioeconomic gradients, which is consistent with previous studies [45, 46]. This could be due to a higher cost related to nutrition quality foods along with low purchasing power of poor households affecting their intention towards a healthy diet [47]. This indicates that the intention to change towards a healthy dietary behavior is highly influenced by the income level of individuals. Therefore, CVD preventive lifestyle interventions should be contextual and need to identify potential barriers and enabling factors to improve intervention effectiveness.

Furthermore, country of birth was associated with intention to a healthy diet, in which participants from non-European origin had a lower intention to change their dietary habit. Previous studies also showed that immigrant-origin groups have poorer dietary habits leading to a higher rate of diet-related NCDs than the native born European populations [48, 49]. A study by Patel and colleagues found that exposure to increased fat intake related to migration could be the possible reason for the disproportionate prevalence of CVD risk factors among immigrants in Britain [50]. This could be due to the cultural difference in dietary habits along with a better access for foods that are high in fat and sugar in the host country [51–53]. Hence, lifestyle interventions targeting dietary change should be culturally adapted to improve reachability and effectiveness on target vulnerable communities.

The present study has the following limitations. First, the sampling and data collection procedure varies between two settings, which could be the reason for a significant discrepancy in the level of knowledge and intention to change. Thus, it is hardly possible to claim this significant difference reflects the difference in population parameters in two settings. Nevertheless, the results mainly indicate that the level of knowledge and risk perception is low in both settings regardless of the variation in the samples. Second, despite districts being selected based on composite vulnerability indices, we did not measure individual vulnerability indices. Thus, future studies recruiting samples based on individual vulnerability indices are recommended. Third, due to lack of individual level socioeconomic factors in Nottingham, the multivariable modeling was limited to data from the Antwerp setting. Hence, the socioeconomic disparities in our study are solely to this setting. Nevertheless, as the two contexts are somewhat comparable, we expect similar socioeconomic disparities in Nottingham as well. Lastly, we did not measure and adjust for physical parameters such as BMI that could affect an individual’s risk perception and intention towards a healthy lifestyle.

## Conclusions

The knowledge towards CVD risk factors and prevention is low in vulnerable communities of Antwerp and Nottingham. Females have a better intention to have a healthy diet whereas males have a higher intention to PA. Level of education is significantly correlated with CVD knowledge and intention to a healthier diet. Household income level also determines intention to change dietary habits. Although there is no difference in knowledge, intention to change habits towards a healthier diet is lower among non-European immigrants. These findings suggest that there are opportunities to reduce the CVD incidence in vulnerable communities of high-income countries through innovative participatory educational strategies to improve knowledge as well as intention to a healthy lifestyle. Furthermore, preventive interventions including coaching should be based on a better understanding of the individuals’ lifestyle condition and cultural beliefs through active engagement of the community and individuals. Future studies are recommended to investigate the longitudinal effect of socioeconomic disparities on each stage of change from precontemplation to contemplation, from contemplation to intention or determination and in turn to a change in actual behavior or action. Furthermore, assessing the moderating effect of socioeconomic status on the impact of CVD preventive intervention is also recommended.

## Supplementary Information

**Additional file 1.** Dutch (Flemish) version of the ABCD questionnaire.

## Data Availability

Data are available from the authors upon reasonable request.

## Abbreviations

ABCD: Attitudes and Beliefs about Cardiovascular Disease
CVD: Cardiovascular disease
DALY: disability adjusted life year
HIC: High income countries
ICC: Intraclass Correlation Coefficient
IQR: Interquartile range
LMIC: Low- and Middle-income countries
MD: Mean difference
MDI: Multiple deprivation index
NCD: Non-communicable Disease
PA: Physical activity
PPS: proportionate to population size
SDI: Socioeconomic Deprivation Index
REDCap: Research Electronic Data Capture
SD: Standard Deviation
VIF: Variance Inflation Factor
