# Supplementary material for: Level of cardiovascular disease knowledge, risk perception and intention towards healthy lifestyle and socioeconomic disparities among adults in vulnerable communities of Belgium and England
Source: BMC Public Health. 2022 Jan 29;22:197. doi: 10.1186/s12889-022-12608-z (PMC8800212; doi:10.1186/s12889-022-12608-z)
Supplement: Supplementary file 1 — Additional file 1. Dutch (Flemish) version of the ABCD questionnaire. [file 12889_2022_12608_MOESM1_ESM.docx]

# Dutch (Flemish) version of the ABCD questionnaire

| **Kennis items**  **(Antwoordopties: 1) Juist, 2) Fout, and 3) Weet het niet)** |
| --- |
| 1. Stress is een van de hoofdoorzaken van hartaanvallen en beroertes. |
| 1. Wandelen en tuinieren zijn activiteiten waarmee je het risico op een hartaanval of hartinfarct kan verminderen. |
| 1. Als je 2,5 uur per week matig fysiek actief bent, vermindert jouw kans op een hartaanval of beroerte. |
| 1. Mensen met diabetes hebben een verhoogd risico op een hartaanval of beroerte. |
| 1. Door je stressniveau te beheersen, kan je je bloeddruk onder controle houden. |
| 1. Het drinken van grote hoeveelheden alcohol, kan jouw cholesterol en andere bloedvetten doen stijgen. |
| 1. Er bestaat 'goede' cholesterol en er bestaat 'slechte' cholesterol. |
| 1. Het voorkomen van hartaandoeningen in de familie is geen risicofactor voor hoge bloeddruk. |
| 1. Mensen die roken hebben een verhoogd risico op hart- en vaatziekten. |
| **Risico perceptie items**  **(Antwoordopties: 1) Helemaal niet eens, 2) Niet eens, 3) Eens, en 4) Helemaal eens** |
| 1. Ik heb het gevoel dat ik ooit in mijn leven aan een hartaanval of beroerte zal lijden. |
| 1. Het is waarschijnlijk dat ik in de toekomst een hartaanval of beroerte zal krijgen |
| 1. Het is mogelijk dat ik binnen nu en tien jaar een hartaanval of beroerte zal krijgen. |
| 1. De kans dat ik binnen nu en tien jaar een hartaanval of beroerte krijg, is groot. |
| 1. Ik zal waarschijnlijk een hartaanval of beroerte krijgen door mijn vroegere en/of huidige levensstijl. |
| 1. Ik maak me geen zorgen dat ik een hartaanval of beroerte zou kunnen krijgen. |
| 1. Ik maak me zorgen omdat er een kans bestaat dat ik in de nabije toekomst een hartaanval of beroerte zal krijgen. |
| **Intentie tot lichamelijke activiteit**  **(Antwoordopties: 1) Helemaal niet eens, 2) Niet eens, 3) Eens, en 4) Helemaal eens** |
| 1. Wanneer ik minstens 5 maal 30 minuten per week aan lichaamsbeweging doe, dan doe ik iets goeds voor de gezondheid van mijn hart. |
| 1. Het is belangrijk voor mij om regelmatig aan lichaamsbeweging te doen. |
| 1. Ik overweeg om minstens 5 maal 30 minuten per week aan lichaamsbeweging te doen. |
| 1. Ik ben van plan, of wil, minstens 5 maal 30 minuten per week aan lichaamsbeweging doen. |
| 1. Ik ben er zeker van dat ik een gezond gewicht kan verkrijgen of behouden door minstens 5 maal 30 minuten per week aan lichaamsbeweging te doen in de komende 2 maanden. |
| 1. Ik overweeg niet om minstens 5 maal 30 minuten per week aan lichaamsbeweging te doen. |
| **Intentie tot Intentie tot eetgewoonte**  **(Antwoordopties: 1) Helemaal niet eens, 2) Niet eens, 3) Eens, en 4) Helemaal eens** |
| 1. Wanneer ik per dag ten minste 5 porties fruit en groenten eet, doe ik iets goeds voor de gezondheid van mijn hart. |
| 1. Het is belangrijk voor mij om dagelijks minstens 5 porties groenten en fruit te eten. |
| 1. Ik overweeg om per dag ten minste 5 porties fruit en groenten te eten. |
| 1. Ik ben van plan, of wil, minstens 5 porties groenten en fruit eten per dag. |
| 1. Ik ben er zeker van dat ik de komende 2 maanden ten minste 5 porties fruit en groenten per dag kan eten. |
| 1. Ik ben er zeker van dat ik een gezond gewicht kan verkrijgen of behouden door per dag minstens 5 porties groenten en fruit te eten in de komende 2 maanden. |
| 1. Ik overweeg niet om per dag ten minste 5 porties fruit en groenten te eten. |

# English version of the ABCD questionnaire

| **Knowledge items**  **(Response options: 1) True, 2) False, and 3) I don’t know)** |
| --- |
| 1. Stress is one of the main causes of heart attacks and strokes. |
| 1. Walking and gardening are considered types of exercise that can lower the risk of having a heart attack or stroke. |
| 1. Moderately intense activity of 2 ½ hours a week will reduce your chances of having a heart attack or stroke. |
| 1. People who have diabetes are at higher risk of having a heart attack or stroke. |
| 1. Managing your stress levels will help you to manage your blood pressure. |
| 1. Drinking large amounts of alcohol can increase your cholesterol and other blood fats. |
| 1. There is 'good' cholesterol and there is 'bad' cholesterol. |
| 1. A family history of heart disease is not a risk factor for high blood pressure. |
| 1. People who smoke have an increased risk of cardiovascular disease. |
| **Risk perception items**  **(Response options: 1) Strongly disagree, 2) Disagree, 3) Agree, and 4) Strongly agree** |
| 1. I have the feeling that someday I will suffer a heart attack or stroke in my life. |
| 1. It is likely that I will have a heart attack or stroke in the future. |
| 1. It is possible that I will have a heart attack or stroke within the next ten years. |
| 1. The chance that I will have a heart attack or stroke within the next ten years is high. |
| 1. I will probably have a heart attack or stroke because of my past and / or current lifestyle behavior. |
| 1. I am not worried that I might have a heart attack or stroke. |
| 1. I am concerned because there is a chance that I will have a heart attack or stroke in the near future. |
| **Intention to physical activity**  **(Response options: 1) Strongly disagree, 2) Disagree, 3) Agree, and 4) Strongly agree** |
| 1. When I exercise for 30 minutes at least 5 times a week, I do something good for the health of my heart. |
| 1. It is important for me to exercise regularly. |
| 1. I am considering exercising 30 minutes for at least 5 times a week. |
| 1. I intend or want to exercise 30 minutes for at least 5 times a week. |
| 1. I am sure that I can gain or maintain a healthy weight by exercising 30 minutes for at least 5 times a week in the next 2 months. |
| 1. I am not considering exercising 30 minutes for at least 5 times a week. |
| **Intention to dietary habit**  **(Response options: 1) Strongly disagree, 2) Disagree, 3) Agree, and 4) Strongly agree** |
| 1. When I eat at least 5 servings of fruit and vegetables a day, I do something good for the health of my heart. |
| 1. It is important for me to eat at least 5 servings of fruit and vegetables every day. |
| 1. I am considering eating at least 5 servings of fruit and vegetables a day. |
| 1. I intend, or want, to eat at least 5 servings of fruit and vegetables a day. |
| 1. I am sure that I can eat at least 5 servings of fruit and vegetables a day for the next 2 months. |
| 1. I am sure that I can gain or maintain a healthy weight by eating at least 5 servings of fruit and vegetables a day in the next 2 months. |
| 1. I am not considering eating at least 5 servings of fruit and vegetables a day. |
